# Spatial Domain Image Fusion with Particle Swarm Optimization and Lightweight AlexNet for Robotic Fish Sensor Fault Diagnosis

**DOI:** 10.3390/biomimetics8060489

**Published:** 2023-10-17

**Authors:** Xuqing Fan, Sai Deng, Zhengxing Wu, Junfeng Fan, Chao Zhou

**Affiliations:** 1 Institute of Automation, Chinese Academy of Sciences, Beijing 100190, China; fanxuqing2021@ia.ac.cn (X.F.);; 2 University of Chinese Academy of Sciences, Beijing 101408, China

**Keywords:** image fusion, lightweight AlexNet, particle swarm optimization, fault diagnosis, robotic fish

## Abstract

Safety and reliability are vital for robotic fish, which can be improved through fault diagnosis. In this study, a method for diagnosing sensor faults is proposed, which involves using Gramian angular field fusion with particle swarm optimization and lightweight AlexNet. Initially, one-dimensional time series sensor signals are converted into two-dimensional images using the Gramian angular field method with sliding window augmentation. Next, weighted fusion methods are employed to combine Gramian angular summation field images and Gramian angular difference field images, allowing for the full utilization of image information. Subsequently, a lightweight AlexNet is developed to extract features and classify fused images for fault diagnosis with fewer parameters and a shorter running time. To improve diagnosis accuracy, the particle swarm optimization algorithm is used to optimize the weighted fusion coefficient. The results indicate that the proposed method achieves a fault diagnosis accuracy of 99.72% when the weighted fusion coefficient is 0.276. These findings demonstrate the effectiveness of the proposed method for diagnosing depth sensor faults in robotic fish.

## 1. Introduction

After undergoing long-term biological evolution and natural selection, fish have developed remarkable abilities to swim rapidly and perform agile maneuvers in complex and dynamic aquatic environments [1]. Taking inspiration from natural fish, robotic fish act as a dedicated underwater vehicle platform offering diverse potential applications, whether in a cooperative or noncooperative manner. These applications include ocean exploration, seabed mapping, water monitoring, underwater pipeline tracking, and more [1,2]. Compared to conventional propeller-driven underwater vehicles, robotic fish possess several favorable characteristics. Firstly, their appearance and movement closely resemble real fish, allowing effective deception and mimicry of the behavior of aquatic organisms. This characteristic facilitates easier access and observation of underwater life while ensuring minimal disturbance and impact on the natural environment during exploration or monitoring. Consequently, data collection becomes more reliable and representative. Secondly, bionic robotic fish exhibit enhanced flexibility and mobility. By mimicking the body shape and movements of real fish, they can navigate quickly through the water and perform a variety of tasks in intricate or confined spaces [3]. Additionally, these robots replicate the streamlined shape and efficient propulsion mechanism of real fish, resulting in superior hydrodynamic performance and significantly improved energy efficiency.

Robotic fish are equipped with numerous and diverse sensors, such as depth sensors, vision sensors, and inertial measurement units, to enable precise perception and intelligent control [4]. However, if the sensors break down during operations, not only do their sensed information become unreliable, but also the entire system may become paralyzed and may even cause safety accidents. To ensure the safe and reliable operation of robotic fish, it is critical to promptly and accurately diagnose sensor faults.

Fault diagnosis is a critical task in various fields, and it can be achieved through different methods, such as signal analysis-based, model-based, and data-driven methods [5]. Recently, there has been a growing interest in intelligent data-driven fault diagnosis methods, driven by the development of deep learning algorithms [6]. Compared to manual extraction, end-to-end deep learning methods have the ability to automatically extract features of the data distribution, resulting in time-saving and efficient utilization of labor resources [7,8,9]. To achieve high precision and fast fault diagnosis, Fang et al. [10] and Chen et al. [11] used one-dimensionality convolutional neural networks (CNN) to extract the multichannel features in order to effectively improve the accuracy of the diagnosis. The former decreased the number of convolution kernels with the reduction in the convolution kernel size and the latter adopted dynamic convolution with separable convolution to classify faults. Liu et al. [12] combined the advantages of long short-term memory (LSTM) network with statistical process analysis to predict the fault of aero-engine bearing and obtained ideal accuracy. Tang et al. [13] proposed signal embedding to solve the problem of transformer application in mechanical vibration signals, which has outstanding performance in terms of diagnostic accuracy under unknown operating conditions in a robustness way. Chen et al. [14] explored the compound fault of industrial robots and proposed an efficient convolutional transformer. The proposed lightweight convolutional transformer network enhanced the meta-learning method to achieve accurate compound fault diagnosis with limited samples.

However, the methods above mainly focus on time domain features, neglecting the spatial domain features. To improve the accuracy of fault diagnosis algorithms, researchers have attempted to convert one-dimensional time series signals into two-dimensional images, and then extract spatial features from the images. For example, Wen et al. [15] reshaped vibration signals into grey images and used LeNet-5 to classify images, leading to significant improvements compared to fault diagnosis based solely on time domain features. Yang et al. [16] adopted the Short Time Fourier Transform (STFT) to transform the signal into the corresponding time-frequency map, which contains abundant feature information. But STFT heavily relies on the window length selected and has significant uncertainty. Xu et al. [17] proposed the generalized S-synchroextracting transform, a new time–frequency post-processing algorithm to address this issue. Xun et al. [18] used the Markov transfer field, which jointly improved deep CNN with a wide first-layer Kernel, improving the sensitivity to spatial features. To further improve fault diagnosis performance, Hou et al. [19] proposed a spatial domain image fusion method. Signals were converted into Gramian angular summation field (GASF) images and Gramian angular difference field (GADF) images using the Gramian angular field (GAF) method, and combined half to half. This method achieved great results for fault location, but whether 0.5 is the optimal weighted combination coefficient needs further discussion. Sun et al. [20] adopted continuous wavelet transform to transform the nonlinear and non-stationary original vibration signal into a time–frequency image, and then used an improved AlexNet model to diagnose faults. Amiri et al. [21] used the recurrence plots method to convert signal to image, and derived the degree of determinism in the signal to detect series arc faults. The results confirm its high accuracy, high speed, and low computing cost.

As for spatial domain features recognition, several CNN-based methods have been proposed for the recognition of spatial domain features, such as AlexNet [20], DenseNet [22], ResNet [23], and so on. Though they have achieved excellent recognition results, they were difficult to apply in practice due to the high time costs and great computing resources. Therefore, scholars conducted research on lightweight network. For instance, Sun et al. [20] replaced the global average pooling (GAP) layer with the fully connected layer, which realizes the improvement of the traditional AlexNet model and the reduction in parameters. Liu et al. [24] replaced large convolution kernels with small convolution kernels to reduce network parameters in AlexNet, which saved model training time significantly. However, these methods targeted three-channel RGB images for local parameter reduction, leaving room for optimizing global parameter reduction for single-channel images.

Although the methods above have significant advantages in terms of accuracy, they require a large amount of computation, and some new methods have started to emerge aiming to reduce the model complexity, such as Inception [25], MobileNet [26], ShuffleNet [27] and so on.

In order to diagnose fault more accurate and faster, the Gramian angular field fusion with particle swarm optimization and lightweight AlexNet (GAFF-PSO-AlexNet) method was proposed to diagnose faults. The main contributions of this article are summarized as follows:

(1) The one-dimensional time series sensor signals are converted into two-dimensional images by using the GAF method. The GASF and GADF images are fused by weighted fusion method to generate Gramian angular field fusion (GAFF) images, and the particle swarm optimization (PSO) algorithm is used to optimize the weighted fusion coefficient.
(2) Lightweight AlexNet is proposed to diagnose six sensor fault types. In order to use fewer parameters and shorter running time, the channels of conventional layer and nodes of fully connected layers are decreased to $\frac{1}{3}$ compared with the original AlexNet.

## 2. Fault Diagnosis Method

### 2.1. Data Preprocessing

#### 2.1.1. Signal to Image

**Figure 1:**
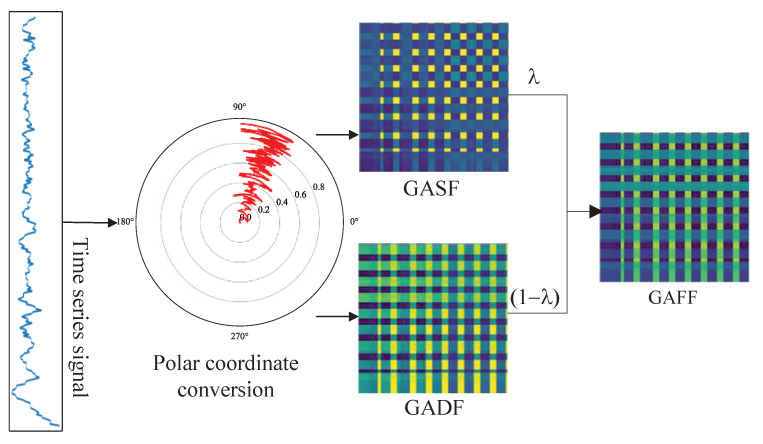
Spatial domain image fusion.

The depth sensor data are one-dimensional time series signals that contain a large amount of time domain information, but it is difficult to extract spatial information directly. To take full advantage of spatial domain information, the GAF method is used to convert one-dimensional time series sensor signals into two-dimensional images, which is shown in Figure 1.

The primary concept of the GAF method is to transform one-dimensional time series signals in the Cartesian coordinate system to the polar coordinate system, followed by using trigonometric functions to create a GAF matrix. This approach eliminates noise in the time series via spatial transformation, and retains time information via vector inner product. The GAF matrix has two types of images: GASF and GADF images. The GASF images are the cosine of the summed angles, while the GADF images are the sine of the subtracted angles. The mathematical representation of this approach can be explained as follows [28]:

We suppose that there is a one-dimensional time series signal denoted as $S=s_{1},s_{2},\ldots ,s_{N}$. Firstly, we normalize *S* by rescaling the values such that it falls under the interval of [−1, 1] with the equation below:

(1) $${\tilde{s}}_{i}=\frac{\left(s_{i}-\max\left(S\right)\right)+\left(s_{i}-\min\left(S\right)\right)}{\max(S)-\min(S)},$$

where ${\tilde{s}}_{i}$ represents the normalized $s_{i}$.

Secondly, rescaled signals can be encoded into polar coordinates. The value of the time series is calculated as the angle and its corresponding timestamp is calculated as the radius. The equation is as follows:

(2) $$\left\{\begin{array}{c} \phi_{i}=\arccos\left(\tilde{s_{i}}\right),-1\leq \tilde{s_{i}}\leq 1,\tilde{s_{i}}\in \tilde{S}\\ r_{i}=\frac{t_{i}}{n},t_{i}\in N \end{array}\right.,$$

where $\phi_{i}$ indicates the polar of the polar coordinate, $r_{i}$ represents the radius of the polar coordinate, $t_{i}$ is the time stamp, $\tilde{S}$ indicates the normalization of *S*, and *n* is a scaling coefficient to regularize the polar coordinate system.

Thirdly, GAF can encode the time series in two different ways. One is GASF, using cosine of the summed angles to mine the correlation between different moment points:

(3) $$\begin{array}{cc} \mathrm{GASF} & =\left(\begin{array}{ccc} \cos\left(\phi_{1}+\phi_{1}\right) & \ldots & \cos\left(\phi_{1}+\phi_{n}\right)\\ \cos\left(\phi_{2}+\phi_{1}\right) & \ldots & \cos\left(\phi_{2}+\phi_{n}\right)\\ \vdots & \vdots & \vdots \\ \vdots & \vdots & \vdots \\ \cos\left(\phi_{n}+\phi_{1}\right) & \ldots & \cos\left(\phi_{n}+\phi_{n}\right) \end{array}\right)\\ \; & ={\tilde{S}}^{T}\cdot \tilde{S}-\sqrt{I-{\tilde{S}}^{2}}\cdot \sqrt{I-{\tilde{S}}^{2}}. \end{array}$$

The GADF images are similar to the GASF images except that the GADF images are constructed using the sine of the subtracted angles as follows:

(4) $$\begin{array}{cc} \mathrm{GASF} & =\left(\begin{array}{ccc} \sin\left(\phi_{1}-\phi_{1}\right) & \ldots & \sin\left(\phi_{1}-\phi_{n}\right)\\ \sin\left(\phi_{2}-\phi_{1}\right) & \ldots & \sin\left(\phi_{2}-\phi_{n}\right)\\ \vdots & \vdots & \vdots \\ \vdots & \vdots & \vdots \\ \sin\left(\phi_{n}-\phi_{1}\right) & \ldots & \sin\left(\phi_{n}-\phi_{n}\right) \end{array}\right)\\ \; & =\sqrt{I-{\tilde{S}}^{2}}\cdot \tilde{S}-{\tilde{S}}^{T}\cdot \sqrt{I-{\tilde{S}}^{2}}. \end{array}$$

The GASF images and the GADF images have two significant advantages. Firstly, polar coordinates contain absolute time series relationships because they convert time-varying signals into angular values. Secondly, the original value and angular information can be preserved in the diagonal value, which ensures that the GAF method retains all the information about the one-dimensional time series sensor signals.

#### 2.1.2. Spatial Domain Image Fusion

In order to leverage the benefits of GASF and GADF images, the fusion of these two images is a natural approach. The weighted fusion method is used to generate GAFF images, which is a transparency fusion technique commonly used in image composition and image matting domains. The fusion process is expressed mathematically as shown in the following equation:

(5) GAFF=λ·GASF+(1−λ)·GADF.

The proposed method involves the use of the GASF and GADF images to generate the GAFF images. The GASF images are considered as the foreground images, with transparency represented by λ, while the GADF images are treated as the background images with a transparency of 1−λ. The range of the transparency value is [0, 1]. By fusing the images at each pixel, the GAFF images contain information of both the GASF and GADF images. When λ=0, the GAFF images only contain the GASF information, while a value of 1 for λ yield only contains the GADF information. The optimal weighted fusion coefficient λ can be determined to achieve the ideal fault diagnosis performance.

### 2.2. Lightweight AlexNet

**Figure 2:**
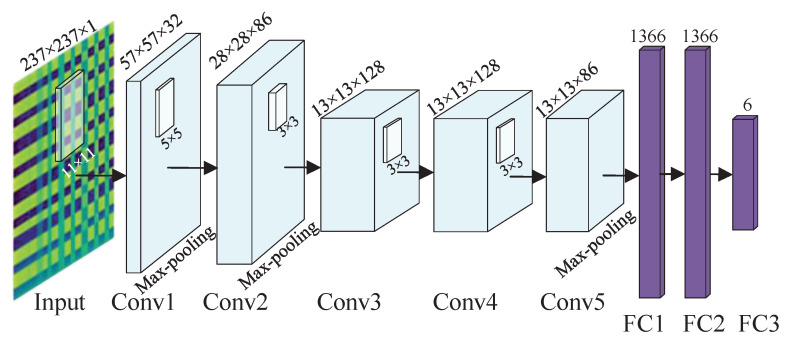
Lightweight AletNet structure.

AlexNet, a model-based CNN which is both deeper and wider, was introduced by Alex Krizhevesky and achieved outstanding performance in the ImageNet challenge for visual object recognition in 2012 [29]. Due to its exceptional ability to perform nonlinear fitting and automatic feature extraction, it has gained significant attention. However, the AlexNet model has numerous parameters that need to be learned, requiring substantial computational resources and extending model training time. To alleviate this complexity, a lightweight version of the AlexNet model, called the lightweight AlexNet, has been proposed, as illustrated in Figure 2. The proposed model aims to maintain efficient classification capabilities for multiple and complex scenes while reducing model complexity.

The lightweight AlexNet is structured similarly to the original AlexNet, consisting of five convolutional layers, three max-pooling layers, and three fully connected layers. The convolutional layers possess linearity and time-shift invariance properties and can extract features at different scales by employing different sizes of convolutional kernels. In this work, kernels of sizes (11×11), (5×5), and (3×3) were selected. Pooling layers, also referred to as downsampling layers, compress the feature map, reduce feature dimensionality, and avoid overfitting without increasing the learned parameters. Furthermore, the fully connected layers in AlexNet use the dropout operation to set the output of hidden layer neurons to 0 when the probability is less than a certain value, which is equivalent to removing some neural nodes to prevent overfitting.

The primary distinction between the lightweight AlexNet and the original AlexNet lies in the reduction in channels for convolutional layers and nodes for fully connected layers by a factor of $\frac{1}{3}$, as indicated in Table 1. In the original AlexNet, the input images consist of red, green and blue three channels, with the corresponding channel numbers for convolutional kernels being 96, 256, and 384, and the number of nodes for fully connected layers being 4096. However, since GAFF images are single-channel grey images, such a large number of channels are not necessary. Consequently, we decreased the channel numbers to 32, 86, and 128, and the nodes to 1066, leading to a decrease in the model parameters.

The complexity of the model can be characterized by two key metrics: space complexity and time complexity. The former is assessed by the total number of parameters in the model, including the weights and biases across all layers, while the latter is reflected in the computational demands of the model, typically quantified as the number of floating point operations (FLOPS) required for training.

For convolutional layers in AlexNet and lightweight AletNet, the parameters and FLOPs can be calculated as follows:

(6) $$\left\{\begin{array}{c} P_{c}=k_{h}\cdot k_{w}\cdot C_{in}\cdot C_{out}+C_{out}\\ F_{c}=2\cdot k_{h}\cdot k_{w}\cdot C_{in}\cdot C_{out}\cdot H\cdot W \end{array}\right.,$$

where $P_{c}$ and $F_{c}$ refer to the parameters and FLOPs of the conventional layer, respectively. $C_{in}$ and $C_{out}$ indicate the channel numbers of the input and output to the conventional layer, while $k_{h}$ and $k_{w}$ represent the height and width of the kernel. Additionally, *H* and *W* denote the height and width of the output feature map.

For fully connected layers in AlexNet and lightweight AletNet, the parameters and FLOPs numbers can be calculated as follows:

(7) $$\left\{\begin{array}{c} P_{l}=L_{in}\cdot L_{out}+L_{out}\\ F_{l}=2L_{in}\cdot L_{out} \end{array}\right.,$$

where $P_{l}$ and $F_{l}$ indicate the parameters and FLOPs of fully connected layer, respectively. $L_{in}$ and $L_{out}$ are channel numbers of input and output to fully connected layer.

For max pooling layers in AlexNet and lightweight AletNet, the parameters are zero and the FLOPs numbers can be calculated as follows:

(8) $$F_{p}=k_{h}\cdot k_{w}\cdot H\cdot W\cdot C_{out}.$$

In order to determine the degree of reduction in total parameters and FLOPs, we define the ratios $R_{P}$ and $R_{F}$ as follows:

(9) $$\left\{\begin{array}{c} R_{P}=\frac{P_{LA}}{P_{A}}\\ R_{F}=\frac{F_{LA}}{F_{A}} \end{array}\right.,$$

where $P_{A}$ and $P_{LA}$ indicate the total parameters of AletNet and lightweight AletNet, and $F_{LA}$ and $F_{A}$ indicate the total FLOPs of above two networks, respectively. As we can see in Table 1, the parameter ratio and the FLOPs ratio of the first conventional layer and the last fully connected layer approximately equal to $\frac{1}{3}$, the parameter ratio and the FLOPs ratio of other conventional layers and the fully connected layers approximately equal to $\frac{1}{9}$, the parameter ratio and the FLOPs ratio of max pooling layers approximately equal to $\frac{1}{9}$, and the total parameter ratio and total FLOPs ratio are approximately equal to $\frac{1}{9}$, which effectively reduces space complexity and time complexity.

However, while bringing advantages, lightweight AlexNet also brings risks associated with reducing the channels and nodes, including, for example, feature representation decreases, information loss and model under-fitting.

### 2.3. Weighted Fusion Coefficient Optimization

To improve the accuracy of fault diagnosis, we propose the utilization of intelligent optimization methods to determine the optimal weighted fusion coefficient. This study explores several heuristic swarm optimization algorithms, including Genetic Algorithm (GA), Ant Colony Optimization (ACO), Whale Optimization Algorithm (WOA), Grey Wolf Optimizer (GWO), and PSO, among others. These algorithms employ iterative computations and evaluation functions to efficiently search for the optimal value, making them well-suited for tackling nonlinear optimization problems.

#### 2.3.1. Optimization Algorithm Selection

According to recent research, it has been demonstrated that ACO, WOA and GWO are specific variants of the PSO algorithm [30,31]. PSO is an evolutionary algorithm inspired by the foraging behavior of bird flocks in search of food. It incorporates mechanisms of individual improvement, population cooperation, and competition. The fundamental concept of this algorithm is to consider particles as individual entities without volume or mass. Each particle possesses two essential attributes: velocity and position. These attributes are continuously adjusted throughout iterations, aiming to converge towards the global optimum of the particle swarm as well as the particle’s historical optimum. By evolving in this manner, the algorithm strives to discover improved values and enhance overall performance.

GA is an optimization technique that emulates the principles of superiority and inferiority in biological evolution. It possesses characteristics such as self-organization, self-adaptation, and easy parallelism. The fundamental concept of GA is to transform the task of finding an optimal solution into a process of crossover and mutation among chromosomal genes. By applying the rule of superiority, the algorithm selects desirable adaptation values while discarding inadequate data. This process of crossover and mutation is repeated to progressively attain superior solutions. GA facilitates local information sharing through chromosome cross-swapping, whereas PSO globally shares information to guide all particles toward the global optimal solution.

In the context of the sensor fault diagnosis problem based on spatial domain image fusion discussed in this paper, our primary focus lies on achieving accurate fault diagnosis. The accuracy of fault diagnosis is directly influenced by the fusion coefficient, making the selection of the optimization algorithm critical for finding the global optimal solution for this coefficient. By maximizing the utilization of global information and minimizing the risk of getting stuck in local optimum, the probability of obtaining a globally optimal solution is enhanced. Considering the PSO algorithm’s advantages in terms of high utilization of global information and low risk of falling into local optimum, it is the preferred choice for this study.

#### 2.3.2. PSO Mathematical Expression

In the context of the particle swarm optimization (PSO) algorithm operating in the real number space, each potential solution within the search space can be conceptualized as an individual particle maneuvering through the hyperdimensional landscape of the given problem [32]. The position of each particle is determined by the vector $x_{i}\in R^{n}$ and its movement by the velocity of the particle $v_{i}\in R^{n}$, as shown in following equations:

(10) $${\vec{x}}_{i}\left(t\right)={\vec{x}}_{i}\left(t-1\right)+{\vec{v}}_{i}\left(t\right),$$

(11) $$\begin{array}{c} {\vec{v}}_{i}\left(t\right)={\vec{v}}_{i}\left(t-1\right)+\varphi_{1}\cdot r_{1}\cdot \left({\vec{p}}_{i}-{\vec{x}}_{i}\left(t-1\right)\right)\ldots \\ +\varphi_{2}\cdot r_{2}\cdot \left({\vec{p}}_{g}-{\vec{x}}_{i}\left(t-1\right)\right), \end{array}$$

where $\varphi_{1}$, $\varphi_{2}$ are two positive numbers and $r_{1}$, $r_{2}$ are two random numbers with uniform distribution in the range of [0, 1]. ${\vec{p}}_{i}$ is a particle’s best position and ${\vec{p}}_{g}$ is a global best position. As we can see, the velocity update equation in Equation (11) has three major components, which represents three properties as follows [33]:

(1) The first component is sometimes referred to as “inertia”, “momentum”, or “habit”. It models the tendency of the particle to continue in the same direction it has been traveling.
(2) The second component of the velocity update equation is a linear attraction towards the best position ever found by the given particle.
(3) The third component of the velocity update equation is a linear attraction towards the best position found by any particle.

### 2.4. Fault Diagnosis Architecture

**Figure 3:**
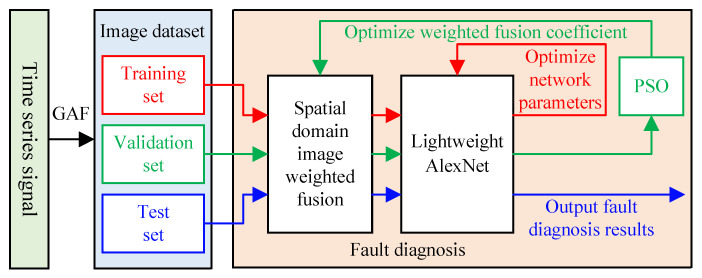
Fault diagnosis architecture.

By synthesizing the strengths of spatial domain image fusion, lightweight AlexNet and PSO, we propose a complete architecture for fault diagnosis, as shown in Figure 3.

The architecture begins with the construction of an image dataset by converting one-dimensional time series signals into two-dimensional spatial domain images using the GAF method. Afterward, the dataset is split into training, validation, and test sets. The training set is employed to optimize the parameters of the lightweight AlexNet network. Then, the validation set data are used to optimize the weighted fusion coefficients using the PSO algorithm. Finally, the test set data are fed into the network parameter fault diagnosis architecture, utilizing the optimal fusion coefficients and trained network parameters, to evaluate the performance of the model.

## 3. Experiment

In order to verify the performance of GAFF-PSO-AlexNet method in practical scenarios, validation experiments were designed on the robotic fish platform, and depth sensor was selected for the research.

### 3.1. Data Collection

**Figure 4:**
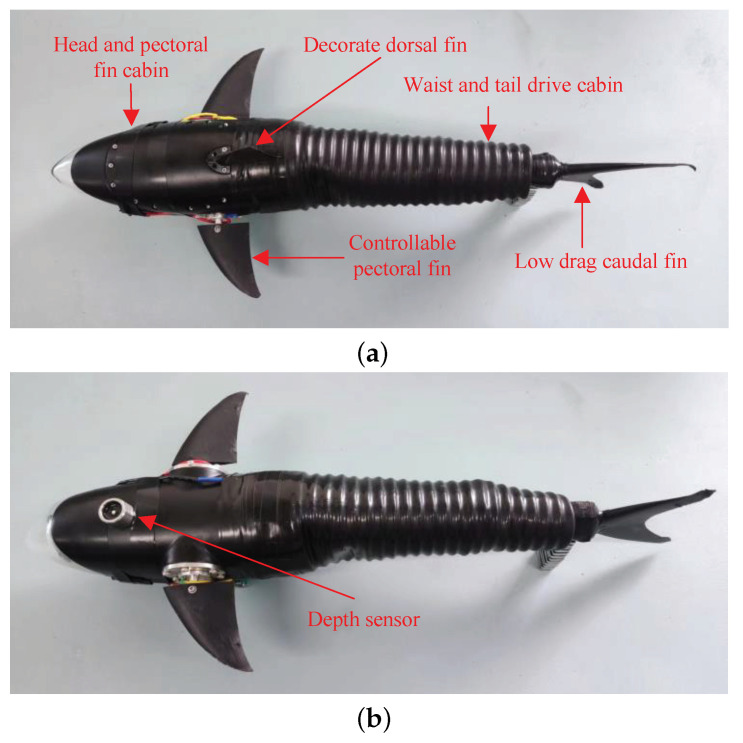
Prototype of the developed robotic fish. (**a**) Top view of the robotic fish; (**b**) Bottom view of the robotic fish.

Our laboratory developed a robotic fish that imitates the structure of a shark in terms of its streamlined shape, which helps to minimize water resistance [34]. Figure 4a depicts the three-link posterior body of the robot, equipped with a lunate caudal fin for thrust generation. The robot is also fitted with a pair of pectoral fins possessing two independent degrees of freedom for orientation and depth adjustment. The fore body of the robot is made of the acrylonitrile–butadiene–styrene (ABS) copolymer, while the posterior body is coated with shin, rendering it as water-resistant as possible. The final prototype of the robot has dimensions of 48.3 cm in length (maximum, including caudal fin), 20.8 cm in width (maximum, including pectoral fins), and 12.5 cm in height (maximum, including dorsal fin), with an approximate weight of 1.35 kg.

An embedded control system based on the STM32F407 micro-controller is developed to enable excellent underwater swimming performance of the robotic fish. The Central Pattern Generator (CPG)-governed control strategy is employed to achieve various shark-like movements, such as forward and backward swimming, turning, diving, and surfacing. The robotic fish is powered by 7.4 V direct current batteries that provide operational flexibility by freeing it from power cable constraints. To achieve intelligent perception and precise control, it is equipped with various sensors such as Inertial Measurement Unit (IMU), depth sensor, camera, and infrared sensor. Among these sensors, the depth sensor is installed on the bottom surface of the robotic fish, as shown in Figure 4b, making it more vulnerable to underwater obstruction collisions than other sensors installed inside the robotic fish. Therefore, this study focuses on fault diagnosis of the depth sensor.

**Figure 5:**
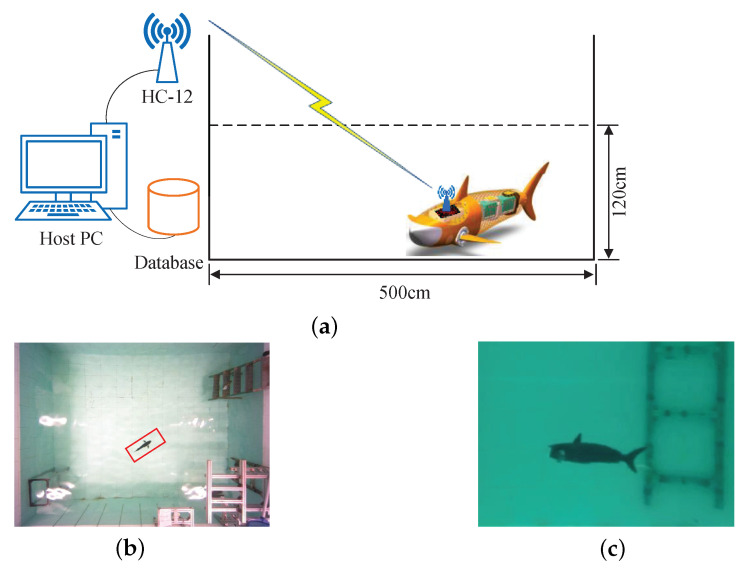
Data collection process and swimming motion of the robotic shark. (**a**) Schematic diagram of data collection; (**b**) Top view of robotic shark swimming in pool under depth control; (**c**) Side view of robotic shark swimming in pool under depth control.

Aquatic experiments are conducted in a laboratory pool measuring 500 cm long, 400 cm wide, and 120 cm high. To collect data automatically, a data collection system based on the HC-12 wireless communication module is designed, as depicted in Figure 5a. The HC-12 module operates at the 433 MHz frequency band and has a high transmitting power, making it suitable for communicating with the robotic shark. The host PC is used for remote control and monitoring of the robotic fish, and the robotic shark is responsible for sensor data collection, swimming motion, and communication with the host PC. The collected sensor information is recorded in a database.

Following the configuration of hardware and software, data are collected from the robotic fish. The process begins with the robot fish in normal operation, and after a period of time, the occurrence of sensor faults is observed. Depth control commands are sent by the host PC, following which the robotic shark moves as per the control law. The top view and side view of the robotic shark during the depth control process are shown in Figure 5b,c, respectively. During this process, the host PC is sending port and the robotic shark is receiving port. Real-time sensor information is recorded by the robotic shark on a Secure Digital (SD) Card. Upon completion of all the motions, the robotic shark sends the information in the SD card to the host PC. The host PC receives sensor data and records them in a database with labels, while the robotic shark is sending port and the host PC is receiving port during this process. In order to minimize the impact of different robotic fish tasks on sensor fault diagnosis, the depth data are used for fault diagnosis by subtracting the target depth value from the sensor’s depth value. Thus, the data collection work is completed.

The depth sensor has a variety of fault types. In the experiment, we only considered six types as shown in Table 2, and some other fault types were not considered due to the limitation of the experimental conditions, such as those arising from poor generation of signals from the robotic fish through to poor transmission of signals, and so on. Our experiment included the normal type and five fault types, namely the depth sensor with no output fault, the depth sensor with intermittent output fault, the depth sensor with jumping output fault, the depth sensor with drifting output fault, and the depth sensor with constant output fault. To avoid quantitative issues with the values and accelerate the convergence of the neural network, the signals were normalized to the range of [0, 1]. Additionally, to eliminate the influence of unbalanced data, an equal number of samples were selected for each type from the collected data. Finally, in order to make full use of collected data, the ratio of 6:2:2 was used to divide the training set, validation set and test set [35]. In practical applications, the data division ratio depends on the specific problem and the size of the data.

The Wilcoxon rank sum test is a nonparametric test method, the contribution of which is to measure the distribution difference between two groups of data samples [36]. Without any special assumptions about the distribution of objective data, the Wilcoxon rank sum test can be applied to some complicated distribution situations. Consequently, the Wilcoxon rank sum test is used to measure the distribution difference between two random types data. The hypothesis $H_{0}$ is proposed that the two types data have the same distribution at the significant level β.

If the hypothesis $H_{0}$ is accepted, it means they are similar in the distribution of the two types of data. In other words, once the hypothesis $H_{0}$ is rejected, it means there is a big difference in the distribution of the two types of data. We randomly selected one data type in each fault data and performed the Wilcoxon rank sum test between two of the six fault data, and the significant level of β=0.005 was achieved. The result of the Wilcoxon rank sum test is shown in Table 3.

As we can see, the Wilcoxon rank sum test value of normal data and other fault data is 0, which indicates that they are more different from each other. The values of F1 and F5, F2 and F5 are all greater than β, which were tested as the same category and are easily misclassified. The Wilcoxon rank sum test value of the F1 fault type and F2 fault type is 0.005, which is in a critical state. The values between the other fault types are less than β, indicating that the data are significantly different from each other and the probability of correct classification is relatively high.

### 3.2. Algorithm Implementation

**Figure 6:**
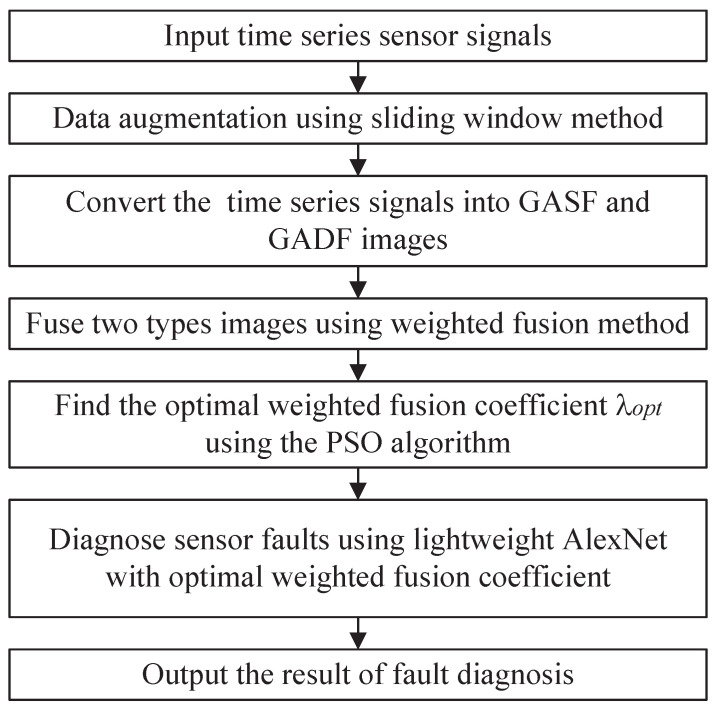
Total flowchart of GAFF-PSO-AlexNet.

After completing the data collection work, the next step is to use the proposed algorithm to diagnose faults. The total fault diagnosis flowchart of spatial domain image fusion with PSO and lightweight AlexNet is shown in Figure 6, which can be divided into the following steps:

Step 1: The time series sensor signals collected from robotic fish in the depth control are inputted to the GAFF-PSO-AlexNet fault diagnosis model.

Step 2: The sliding window method is used to segment the original signal into a series of equal-sized sub-signals and regard each sub-signal as one sample, achieving the effect of data augmentation.

Step 3: The time series sensor signals are converted into GASF images and GADF images using the GAF method.

Step 4:The GASF and GADF images are fused using Equation (5) to make full use of the information in two types of images.

Step 5: The PSO algorithm is adopted to find the optimal weighted fusion coefficient $\lambda_{opt}$.

Step 6: With the optimal weighted fusion coefficient, lightweight AlexNet is used to diagnose fault types in the depth sensor.

Step 7: The result of fault diagnosis is output, including confusion matrix, accuracy, precision rate, recall rate and F1-Score.

In teh above Step 4, the PSO algorithm is employed to obtain the optimal weighted fusion coefficient. In this process, the accuracy of the validation set in the lightweight AlexNet is utilized as the fitness function, and the parameter to be optimized is the weighted fusion coefficient. To perform the optimization, a pack of three particles is selected and the maximum number of iterations is set to 60. The specific steps of the optimization process are presented in Algorithm 1.

The proposed fault diagnosis method was implemented in a Python environment on a computer equipped with an Intel 3.8 GHz Core i7-10700K CPU and NVIDIA RTX 3060 Ti GPU with a memory capacity of 8 GB. The Pytorch framework was utilized for training, validating and testing the GAFF-PSO-AlexNet network.
**Algorithm 1** Framework of the PSO algorithm optimizing weighted fusion coefficient**Input:** the maximum number of iterations *N*; the number of particles *n*; the weighted coefficient upper bound $\lambda_{max}$ and lower bound $\lambda_{min}$**Output:** the optimal weighted coefficient $\lambda_{opt}$  1:Initialize the parameters $\varphi_{1}$, $\varphi_{2}$, $r_{1}$, and $r_{2}$  2:**for** each particle *i*  3:   Initialize position $X_{i}$ and velocity $V_{i}$ for particle *i*  4:   Evaluate particle *i* and set $p_{i}=X_{i}$  5:**end for**  6:$p_{g}$ = min $p_{i}$  7:**while** not stop  8:   **for** i = 1 to N  9:      Update the position and velocity of particle *i*10:      Evaluate particle *i*11:      **if** fit($X_{i}$)<fit($p_{i}$)12:         $p_{i}=X_{i}$13:      **if** fit($p_{i}$)<fit($p_{g}$)14:         $p_{g}=p_{i}$15:   **end for**16:**end while**17:Save optimal weighted fusion coefficients $\lambda_{opt}$ = $p_{g}$18:**return** $\lambda_{opt}$

## 4. Result Analysis

### 4.1. Fault Diagnosis Performance

**Figure 7:**
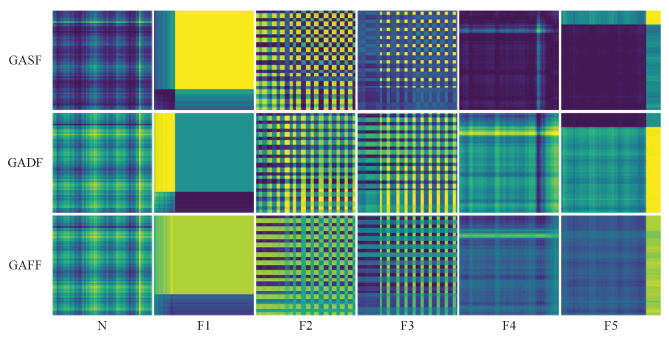
Result of signals converted into images using the weighted fusion method.

With the GAF method and the weighted fusion method, GASF images, GADF images, and GAFF images can be generated as shown in Figure 7. As we can see, GAFF images fuse the information of two types of images which enhanced expression of features. For example, in the F2 fault type, horizontal and vertical stripes are evident in the left half of the GASF and GADF images, and in the GAFF image, clear horizontal stripe features are enhanced and irrelevant vertical stripes are canceled out, similar to the function of feature filtering. In particular, the F2 type and F3 type have similar texture features, and the F1 type and F5 type have similar block characteristics, which bring challenges and attract attention for subsequent precise fault diagnosis.

**Figure 8:**
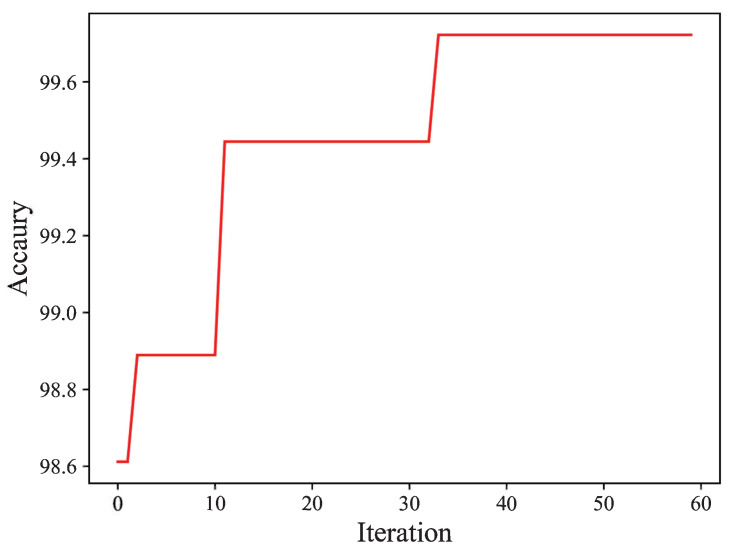
The prediction accuracy with the PSO algorithm.

The PSO algorithm obtains the results of the optimal parameter by preserving the position of the particle with the best fitness. In Figure 8, as we can see, the prediction accuracy improves when the PSO iteration increases. When PSO iterates to the 34th iteration, the optimal solution is found and the algorithm converges to the best classification accuracy; the optimal weighted fusion coefficient is 0.276. Then, the test set data are input with the optimal weighted fusion coefficient, and the fault classification accuracy of robotic fish depth sensor is 99.72%.

**Figure 9:**
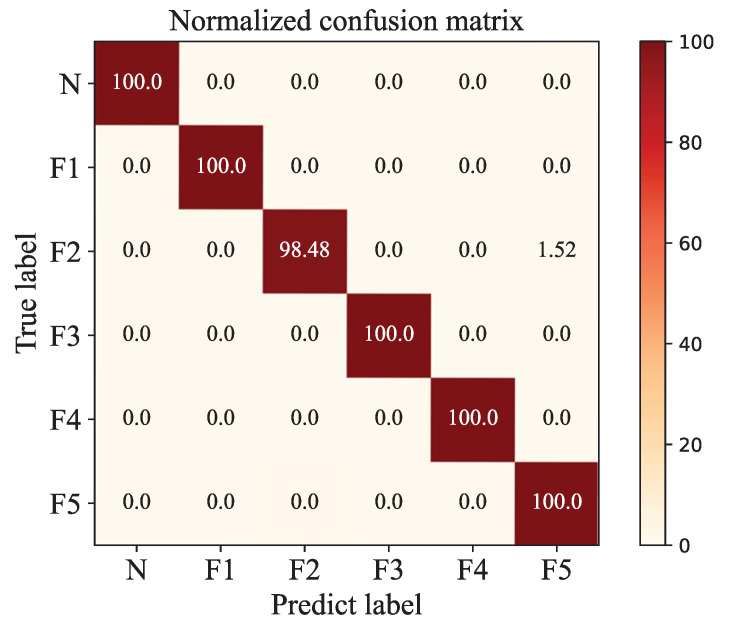
The confusion matrix with the optimal weighted fusion coefficient.

The confusion matrix is a visualization method to represent the accuracy of a model. The confusion of GAFF-PSO-AlexNet is shown in Figure 9.

Each column of the confusion matrix represents the prediction category and each row represents the true attribution category. The shades of the legend color represent the accuracy; the diagonal and non-diagonal lines, respectively, represent the classification accuracy rate and the misclassification rate. As we can see, the fault types are all correctly classified, except the no output faults, 1.52% data are misclassified as the constant output fault.

An ideal fault diagnosis method should have high rate and high precision rate. Recall rate is used to evaluate the coverage of all targets to be classified by the classifier, i.e., to assess whether there are any missing alarms, and precision rate indicates how many of the samples predicted to be a certain sample are really such samples, i.e., to assess whether there is a false alarm. To achieve a balance between precision rate and recall rate, the F1-Score is adopted to measure the classification performance of the model, which can be calculated as follows:

(12) $$F1\mathrm{-Score}=2\times \frac{\mathrm{Precision}\cdot \mathrm{Recall}}{\mathrm{Precision}+\mathrm{Recall}}.$$

In Table 4, as we can see, normal type and three fault types have a 100% F1-Score. Not only do they have no missing alarm, but they also have no false alarm. It is proven that the model has very good classification effect on the above four sensor types. F2 has a missing alarm and F5 has a false alarm since some F2 samples were misclassified to F5 types, which is consistent with the results of the Wilcoxon rank sum test.

**Figure 10:**
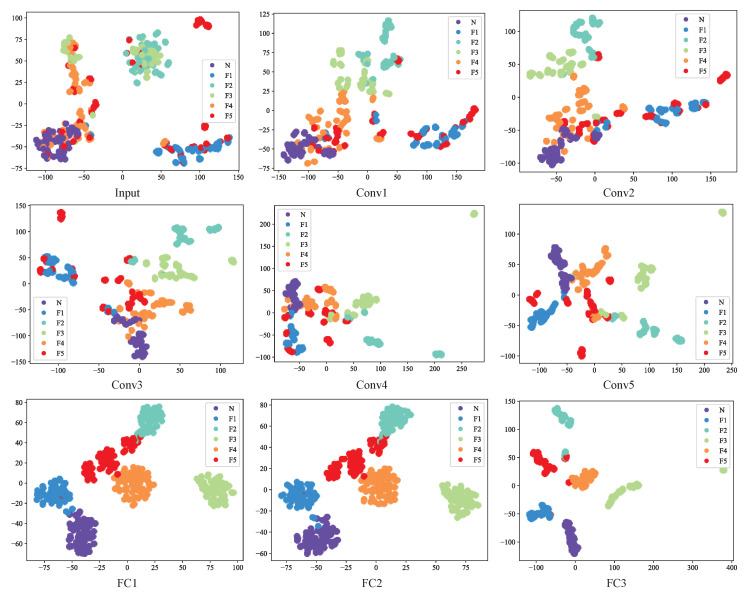
Visualization of the features.

In order to illustrate the superior discriminability of the features acquired by the GAFF-PSO-AlexNet algorithm, we employed the T-distributed Stochastic Neighbor Embedding (T-SNE) algorithm [37], which is a dimensionality reduction method that can reduce the abstract features in a high dimensional space to a low dimensional space. The different layers of features were reduced to visualize the features in the 2D embedding space, as shown in Figure 10. As we can see, at the input layer, data from different fault types have a significant overlap, making it difficult to distinguish between fault categories. As features are extracted layer by layer, different types of fault data gradually separate, and data from the same type of fault gradually cluster together. In the third fully connected layer, it can be clearly seen that different types of fault data were separated in a two-dimensional space.

To further verify the effectiveness of our methods, we constructed six fault diagnosis methods based on ResNet, DenseNet, ShuffleNet, MobileNet, and Inception feature extraction models and compared these methods with our method in terms of average test accuracy, precision, recall and F1-Score on the robotic fish dataset. Table 5 shows the performance comparison experimental results. As we can see, our method has the highest accuracy, precision, recall and F1-Score, proving the superiority of the approach. ShuffleNet and MobileNet have fewer parameters and FLOPs, but fault diagnosis accuracy is lower than that of lightweight AlexNet.

### 4.2. Comparison of AlexNet and Lightweight AlexNet

In order to evaluate the impact of lightweight AlexNet, we conducted a series of experiments to compare the fault diagnosis accuracy and fault diagnosis time. Specifically, we investigated the fusion coefficients in the range of [0, 1] with intervals of 0.05, and analyzed the accuracy, training time, and loss function drop curves of both AlexNet and lightweight AlexNet.

**Figure 11:**
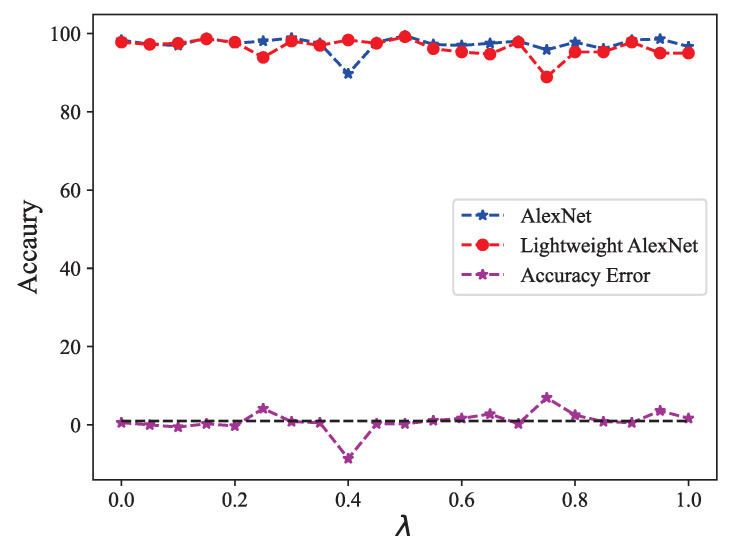
Accuracy comparison between AlexNet and lightweight AlexNet.

The effectiveness of fault diagnosis is primarily evaluated based on the accuracy of correctly classified samples, and achieving high fault diagnosis accuracy is a crucial objective. In this study, we conducted a comparative analysis of accuracy at different weighted fusion coefficients, as illustrated in Figure 11. The average accuracy of AlexNet and lightweight AlexNet were determined to be 97.30% and 96.38%, respectively, while their maximum accuracy were reported as 99.44% and 99.17%, correspondingly. As we can see, lightweight AlexNet has reduced accuracy compared to AlexNet, which is a structural risk due to the reduced number of convolutional kernel channels and the reduced number of nodes in the fully connected layer. Fortunately, the accuracy reduction is not great. However, the lightweight AlexNet exhibited a much smaller model size and faster computation speed, making it a more efficient alternative for fault diagnosis in practical scenarios.

**Figure 12:**
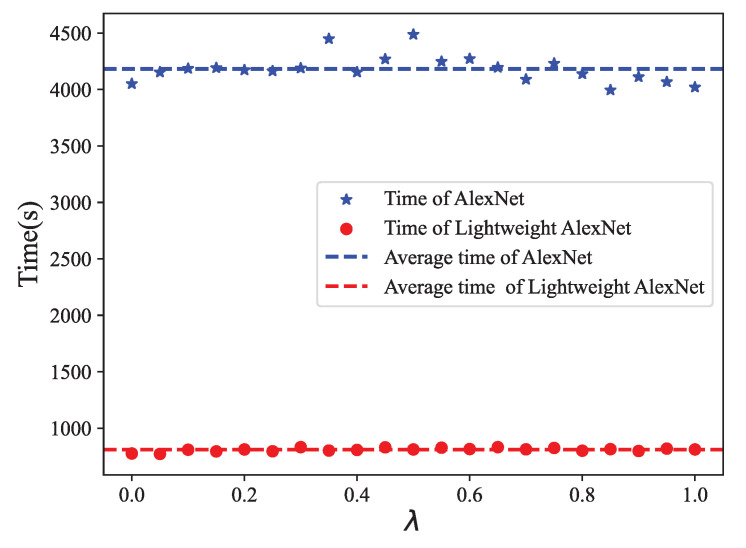
Time comparison between AlexNet and lightweight AlexNet.

In addition, we conducted a comprehensive analysis of the training time required for AlexNet and lightweight AlexNet, both trained for 60 epochs. The experimental results, as demonstrated in Figure 12, indicated that the mean running time for AlexNet and lightweight AlexNet were 4182 s and 809 s, respectively. This substantial decrease in running time highlights the time-saving benefits of employing lightweight AlexNet. Despite the fact that the time complexity was reduced by a factor of $\frac{1}{9}$, as previously mentioned, the actual training time only decreased by approximately $\frac{1}{5}$. One possible explanation is that the actual running time is influenced not only by the computational load but also by the computer’s read/write speed.

**Figure 13:**
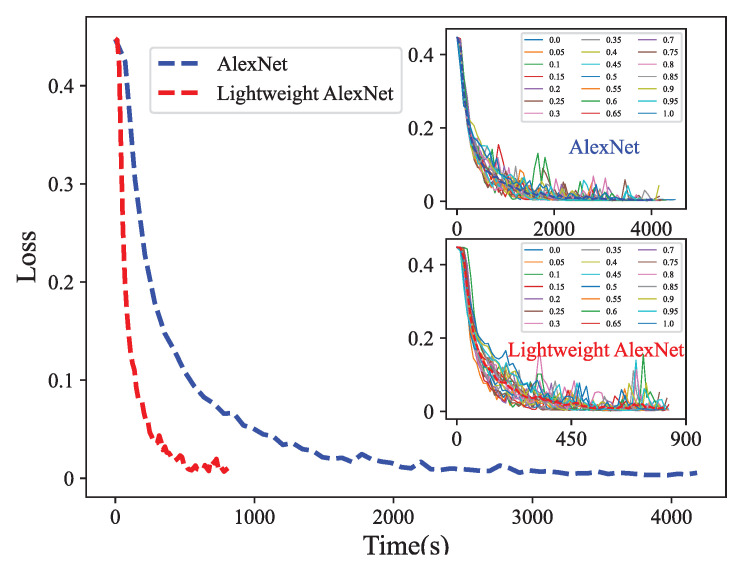
Loss comparison between AlexNet and lightweight AlexNet.

Furthermore, we carried out an in-depth investigation of the performance of the proposed fault diagnosis approach by comparing the error losses of AlexNet and lightweight AlexNet, as depicted in Figure 13. The loss gradually reduces and reaches a near-zero value with an increasing number of training sessions until convergence is achieved. The experimental results demonstrated that lightweight AlexNet achieved significantly faster convergence than AlexNet, thus confirming the efficiency of the algorithm.

In summary, the proposed lightweight AlexNet algorithm demonstrates its efficiency with faster convergence speed. Despite its minor drawback in accuracy compared to the traditional AlexNet, the PSO algorithm can be utilized to search for the optimal fusion coefficient, compensating for the trade-off between accuracy and efficiency.

## 5. Conclusions

In this article, the GAFF-PSO-AlexNet method-based spatial domain image fusion with PSO and lightweight AlexNet was proposed for robotic fish sensor faults to improve classification accuracy and decrease diagnose time. The main contributions of this study are summarized as follows. (i) The one-dimensional time series sensor signals are converted into two-dimensional images by using the GAF method, GASF and GADF images are fused by the weighted fusion method to generate GAFF images, and the PSO algorithm is used to optimize the weighted fusion coefficient. (ii) Lightweight AlexNet is proposed to diagnose fault types with lower time complexity and space complexity. These results suggest that robotic fish sensor faults may be diagnosed with fewer parameters and shorter running time, and the great potential of the proposed GAFF-PSO-AlexNet method in the data-driven fault diagnosis field is shown.

As future work, more fault types need to be explored and the risks of the lightweight AlexNet model need to be quantitatively analyzed. In addition, we are interested in studying the deployment of the algorithms inside bionic robotic fish for real-time fault diagnosis to improve its practicality, detecting the time of fault occurred and exploring to diagnose fault in dynamic variable working conditions.

## Figures and Tables

**Table 1:** Comparison of AlexNet and lightweight AlexNet.

Layer	AlexNet			Lightweight AlexNet			$R_{P}$	$R_{F}$
	**Channel** **Nodes**	**Parameters**	**FLOPs**	**Channel** **Nodes**	**Parameters**	**FLOPs**		
Input	1	-	-	1	-	-	-	-
Conv1	96	11,712	75,480,768	32	3904	25,160,256	0.333	0.333
Max-pooling	96	0	677,376	32	0	225,792	-	0.333
Conv2	256	614,656	963,379,200	86	68,886	107,878,400	0.112	0.112
Max-pooling	256	0	389,376	86	0	130,806	-	0.336
Conv3	384	885,120	299,040,768	128	99,200	33,486,336	0.112	0.112
Conv4	384	1,327,488	448,561,152	128	147,584	49,840,128	0.111	0.111
Conv5	256	884,992	299,040,768	86	99,158	33,486,336	0.112	0.112
Max-pooling	256	0	82,944	86	0	27,864	-	0.336
FC1	4096	37,752,832	75,497,472	1366	4,230,502	8,458,272	0.112	0.112
FC2	4096	16,781,312	33,554,432	1366	1,867,322	3,731,912	0.111	0.111
FC3	6	24,582	49,152	6	8202	16,392	0.334	0.333
Total	-	58,282,694	2,195,753,408	-	6,524,758	262,442,494	0.112	0.120

**Table 2:** Sensor fault conditions.

	Label	Description
1	N	Depth sensor is normal
2	F1	Depth sensor has no output fault
3	F2	Depth sensor has intermittent output fault
4	F3	Depth sensor has jumping output fault
5	F4	Depth sensor has drifting output fault
6	F5	Depth sensor has constant output fault

**Table 3:** Result of the Wilcoxon rank sum test.

	N	F1	F2	F3	F4	F5
N	1	0	0	0	0	0
F1	0	1	0.0050	0	0.0021	0.1327
F2	0	0.0050	1	0	0.0251	0.0222
F3	0	0	0	1	0	0
F4	0	0.0021	0.0251	0	1	0
F5	0	0.1327	0.0222	0	0	1

**Table 4:** Fault diagnosis result with the optimal weighted fusion coefficient.

	N	F1	F2	F3	F4	F5	All
Precision rate (%)	100	100	100	100	100	98.04	99.67
Recall rate (%)	100	100	98.48	100	100	100	99.74
F1-Score (%)	100	100	99.23	100	100	99.01	99.71

**Table 5:** Performance comparison of different models with model parameters, FLOPs, accuracy, recall and F1-Score.

Model	Params (M)	FLOPs (G)	Acc (%)	Pre (%)	Rec (%)	F1-Score (%)
ResNet	23.51	145.17	98.89	98.74	98.86	98.79
DenseNet	6.95	97.92	98.33	98.20	98.41	98.24
ShuffleNet	1.26	0.34	94.72	94.61	95.18	94.73
MobileNet	1.68	0.14	93.89	93.73	93.68	93.64
Inception	41.15	14.48	95.56	95.31	95.56	95.35
Ours	6.52	0.26	99.72	99.67	99.74	99.71

## Data Availability

Not applicable.
